# Enhancing Schools’ Development Activities on Inclusive Education Through In-service Training Course for School Teams: A Case Study

**DOI:** 10.3389/fpsyg.2022.824620

**Published:** 2022-05-27

**Authors:** Tiina Kivirand, Äli Leijen, Liina Lepp

**Affiliations:** Institute of Education, University of Tartu, Tartu, Estonia

**Keywords:** inclusive education, in-service training, school teams, schools’ development process, factors affecting

## Abstract

Most countries face the challenge of reconstructing their education systems to ensure equitable quality education for all children in inclusive settings. This challenge is also relevant in Estonia, the context of this study. A long-term in-service training course for school teams (school leaders, support specialists, and teachers) was developed and implemented in Estonia. The main goal of the training course was to develop attitudes, skills, and knowledge of school staff about the concept and meaning of inclusive education (IE) and the effective implementation through inclusive school development strategies. The aim of the current study was to find out how the in-service training course for school teams influences system-wide changes in the implementation of IE at the school level and what factors affect it. Purposeful sampling (two schools) was used, and the qualitative thematic case study research method was chosen to find answers to the research questions. Data were collected from school policy documents, homework assignments of the training course, semi-structured interviews in the middle and at the end of the training course, open-ended questionnaires at the end of the training course, and researcher diary. The results showed that the in-service training course for school teams enhanced cultural and structural changes at the school level. These changes were influenced by factors such as leadership, collaboration, commitment, and contribution of different parties, system-wide approach, resources, and external expertise. The implications of these findings are discussed further in the paper.

## Introduction

Inclusive education (IE) as a human right (United Nations, 2006) has been ideologically accepted in most countries currently. Nevertheless, many countries are still making efforts restructuring their education systems to provide high-quality education for all learners in inclusive settings. This is complicated by the fact that education systems of different countries are based on deep rooted historical and cultural specificities (Ainscow and Miles, 2008). However, profound changes in the education system require a fundamental transformation of key aspects, ways of thinking, and practices in education. Thus, policymaking, teacher education, school management, and cooperation between different school stakeholders need to change (Arcidiacono and Baucal, 2020).

The literature reviews show that inclusive school development has focused primarily on teachers’ readiness to cope with special educational needs students (SEN) in an inclusive classroom (Van Mieghem et al., 2018; Hansen et al., 2020). Teachers’ knowledge and skills play an important role in implementing inclusive classroom practice. As the implementation of IE is a very complex and multifaceted process (Mitchell, 2015; Schuelka and Engsig, 2020), there are many factors at different levels of education systems that influence a meaningful implementation of IE. The OECD report (OECD, 2003) emphasizes the principle that teaching SEN students is a matter a whole school, not just individual teachers. Ainscow and Miles (2008) have pointed out that, in addition to what is happening at the class level, a school culture and the commitment of all school staff members are equally important. This complex and multifaceted act requires consciously targeted effort and particular ways of leading (Carter and Abawi, 2018). Ainscow and Sandill (2010) emphasize that cultural changes in the workplace affect how teachers view their work and students. Additionally, school policy that support school-wide structural changes is equally important (Hadfield and Ainscow, 2018; Ainscow, 2020). In order to design inclusive schools, the key capacity building strategy is enhancing cross-professional collaboration (Hansen et al., 2020). A school-wide training approach, collaboration between teachers and support professionals, collaboration and support from school leaders and resource centers, including universities, promotes the implementation of different characteristics of inclusive education at the school level (Harris and Jones, 2017; Juma et al., 2017). Bjørnsrud and Nilsen (2019) have pointed out that collective learning in teams paves the way for joint planning with preparation, a common language, observation in the classroom, and new ideas with actions for pupils’ learning. Moreover, the need for research on how to support and advise schools in developing the organization, in collaboration with researchers and practitioners, has been highlighted (Grima-Farrell and Mcdonagh, 2011). Therefore, in addition to teachers’ pre- and in-service training courses, in-service training for school teams of different professionals (teachers, support specialists, school leaders) could help to address these complex challenges.

In this paper, we report on a study that was conducted in Estonia where inclusive education has become an important field of research (see, e.g., Leijen et al., 2021; Pedaste et al., 2021). Moreover, the Teaching and Learning International Survey (TALIS) 2018 report emphasized that, in Estonia, professional development of both teachers and school leaders regarding the successful implementing of IE needs to be enhanced (OECD, 2020). Consequently, developing the teaching quality of IE curriculums at teacher training universities in Estonia has become one of the key priorities. Studies conducted in Estonia have shown that there is a need for different kind of training courses. For example, courses for teachers to develop the competencies needed to teach students with different abilities, training for support professionals on their changed role in implementing IE, but also training for school leaders to structure the inclusive school development process (Kivirand et al., 2020). Therefore, attention has been paid to composing new training courses at the two main teacher training universities in Estonia. In addition to modernizing initial and in-service training courses for teachers on specific topics of IE, an in-service training program was designed for school teams, involving all key actors at school level who play an important role in the meaningful implementation of IE. More precisely, a long-term (60 ECTS) in-service training program on IE was designed, which included a separate course for teachers (24 ECTS), joint courses for teachers and support specialists (26 ECTS), and a joint course for school teams, i.e., teachers, support specialists, and school leaders (10 ECTS; see Kivirand et al., 2021). The main goal of the school teams training course was to develop (1) positive attitudes toward IE and (2) skills and knowledge about the concept and meaning of IE and its effective implementation through inclusive school development strategies. The general principles of the training course were to link theory to practice and raise schools’ capacities to implement IE during the training sessions and designing long-term development activities. We took into consideration that reconstructing the school culture and practice on IE is a very multifaceted and long-term process. To support schools’ development through in-service training, it is important to address all relevant topics in a coherent and cyclical way over a longer-term period. The training was conducted over a period of 1.5 years (for further information see Kivirand et al., 2021).

In this paper, we will explore how the long-term in-service training course for school teams (teachers, support specialists, and school leaders) influenced schools’ development activities in the implementation of IE and what factors affected the implementation from the perspective of the school teams. In the following section, we will introduce the rationale and the theoretical background of the developed course and present the research questions of the study.

## Professional Development of Staff Members on IE

Effective implementation of IE is a multi-faceted endeavor that requires the involvement of all those involved in the school, and above all motivated teachers and a positive attitude to IE (Kaur et al., 2015). So far, teacher education in IE has often focused on increasing teacher-specific didactical competences to cope with children with SEN. It has been stressed that professional development for teachers should pay more attention to build on collaboration and collegial interactions (Mangope and Mukhopadhyay, 2015). According to Forlin and Sin (2017), the development of teacher competencies, as a curriculum for professional learning, requires a number of key principles, including:

- engaging teachers, leaders, and other stakeholders in dialogue regarding which competencies are required;
- developing a vision for professional learning that is integrated into system-wide; and
- whole-school planning.

The sense of a cohesive school community, cooperation between teachers, and support professionals plays an important role in the implementation of meaningful and child-centered IE (Engelbrecht et al., 2017). Evidence shows that many countries face the challenge of how special needs educators could support teachers in inclusive classrooms (Florian, 2019). The transition from integration to inclusion requires a relevant conceptual change for modifying the role of the support teacher with regard to implementing inclusion. In addition to the traditional individual support for children with special needs, there is an increasing role for support professionals in supporting, advising, and collaborating in teaching (Perez et al., 2017). The content of teachers’ and special educators’ training has frequently focused on how to differentiate teaching of SEN students in the mainstream schools rather than on working with all students in an inclusive classroom.

In addition, school leaders play a critical role in creating conditions that positively impact school performance in inclusive practice (Ainscow and Sandill, 2010; Al-Mahdy and Emam, 2017; Amin and Yasin, 2018). They must prioritize equity and excellence for all through their decision-making which affects learner groupings, staff allocation, access to curriculum and accreditation opportunities, and resource allocation (Harris and Jones, 2017; European Agency for Special Needs and Inclusive Education, 2019). School leaders should take a leading role in promoting positive attitudes toward IE and innovation processes when applying inclusive education in everyday practice (Urton et al., 2014). In addition, Skoglund and Stäcker (2016) emphasize that main tasks of school leaders are to set directions for staff and organizational development. Therefore, educational leaders’ values, beliefs, and perceptions toward inclusive education have a large impact on how other stakeholders view inclusion (Cherkowski and Ragoonaden, 2016; Al-Mahdy and Emam, 2017). Schools are successful and provide high-quality education to all students if school leaders themselves enact the school with an inclusive vision and values, while motivating the entire staff to apply an inclusive approach (Schuelka et al., 2018; Kivirand et al., 2021). Studies have shown that school leaders principally value the philosophy of inclusion (Bayrakci et al., 2017; Murphy, 2018), but the problems are reflected in their knowledge, skills, and leadership styles of how to design inclusive organizations (Amin and Yasin, 2018; Carter and Abawi, 2018).

Therefore, in order to succeed in the whole-school system-wide development activities in the field of IE, the professional development and cooperation of all parties is important. An in-service training course for school teams could provide a good opportunity to raise capabilities of all school level parties and opportunities to enhance research-based collaboration between schools and universities (Kivirand et al., 2021). In the following section, we will look at what theoretical starting points we used as a basis for designing in-service training for the school teams (teachers, support specialists, and school leaders).

## Designing the In-Service Training Course for School Teams

Kinsella, 2020 emphasizes that ensuring high-quality education for all children in an inclusive classroom (including children with SEN) depends primarily on the extent to which the entire school staff pays attention to the development of the organization. Changes in the whole school culture and politics require a reflective practice of both the individual and the entire staff, and the key to the success of the collaborator’s problem-solving is team-learning. Hereby it must be considered that the education system as a whole and the school as an organization is a very complex multi-layered socio-cultural system. Thus, development activities must consider many different characteristics that cover all levels of this ecosystem (Haug, 2020; Kinsella, 2020; Schuelka and Engsig, 2020). Therefore, we based the design of the in-service training course for school teams on the ecosystem model for supporting IE developed by the European Agency for Special Needs and Inclusive Education (EASNIE) that was previously developed based on Bronfenbrenner’s ecological system model (European Agency for Special Needs and Inclusive Education, 2017). More specifically, according to this model’s key indicators from meso-system (school level), like leadership, continuum of support, collaboration, professionalism of staff, ethic for everybody and family involvement, were combined with the exo-system around the school, i.e., community commitment and working together with other professionals outside of schools. Finally, macro-system indicators, like state legislation and policy, governance and funding, monitoring and quality assurance, were also taken into account.

The main goal of the school teams training course was to raise the school staff’s awareness about the concept and meaning of IE and its effective implementation through inclusive school development strategies. Schools were first introduced to IE principles and following they analyzed their specific context and planned developmental activities related to IE based on the need of their schools. The elements of three necessary dimensions, like creating inclusive cultures, producing inclusive policies, and exploring inclusive practices, described in the guidebook *Index for inclusion* (Booth and Ainscow, 2002), were used. Although this document has been widely used in many countries, it was considered that the different models developed cannot be replicated one by one, but the local context must be taken into account (Loreman, 2014). Thus, the indicators and questionnaires described in the above-mentioned document were partially used and adapted to the Estonian context. For example, indicators of school culture were translated and mapping of the schools’ contexts in this dimension. An additional source used in the training course was the self-assessment questionnaire addressing key issues at classroom and organization level developed by EASNIE (European Agency for Special Needs and Inclusive Education, 2017). This instrument supported schools to assess the situation with regard to students and school staff, partnership and collaboration, and the role of school leaders.

Finally, a co-creative approach in designing the training course was used to ensure the topics we chose for the training course for school teams made sense and were meaningful for teachers, support specialists, and school leaders. An initial outline of the training course was introduced to and discussed with the participants before the training course. For example, schools expressed the view that the training should address issues of how to work together to set common goals for meaningful implementation of IE, how to create support systems for both students, and teachers to ensure effective teaching in an inclusive classroom. At the same time, the participants’ expectation was that the joint training of the different schools will preserve the autonomy and contextual specificity of each school. According to Vyas et al. (2014), a multi-disciplinary co-creation in the designing process can lead to harmonious work where the insight and previous experiences of the participants provide useful input to the practical research framework.

Based on the abovementioned theoretical framework, the following topics of the training course for school teams (10 ECTS) were identified: vision and school culture, legislative framework and school policy, learning environment and resources, professionalism of staff, collaboration, and quality assurance (see also Kivirand et al., 2021). The training course was divided into nine sessions with 60 academic hours contact training and 200 h independent or group activities. The aim of the current study is to explore how long-term in-service training for school teams (teachers, support specialists, school leaders) influenced schools’ development activities in the implementation of IE and what factors affected it based on the school teams’ perspective.

The following research questions were set:

1. What development activities were carried out during the in-service training course to implement IE at the schools?
2. What factors affected the development activities planned and carried out in the implementation of IE?

## Methodology

An exploratory case study approach was used as it enabled to answer the questions “what” development activities were carried out during the training course to implement IE at the schools and explore “why” or “how” these phenomena appeared in the context these were situated (Baxter and Jack, 2008).

### Selection of Cases

Purposeful sampling was used in which data are collected from people who can best inform the researcher about the research problem under the examination (Creswell, 2007). Two participating schools (out of four) were selected as cases for the current study following these criteria (see also Table 1):

1. Clear initiative from the school to participate in the training course with the aim to carry out school development activities in the field of IE;
2. Students with and without SEN in the area of their school residence study in school;
3. Schools with similar numbers of students in the level of compulsory education managed by the same municipality;
4. Participation of all school levels’ key stakeholders in implementing inclusive education, i.e., school principal, support specialist in the role of the special needs education coordinator (SENCO) and teachers.

**Table 1:** Background data of study participants.

	The whole number of students in the level of compulsory education	The percent of SEN students	The number of special classes	Team members		
				Teachers	Support specialists	School leaders
Case No.1	500–560	30%, of which 78% with additional general support out of classroom, 16% with intensified support and 6% with special support	None	Four subject teachers who teach in grades 4–9	One special educator in the role of SENCO	One school principal, working experience as a school leader at the beginning of the training 2 years
Case No. 2	500–560	26% of which 75% with additional general support out of classroom, 13% with intensified support and 12% with special support	Nine small groups for SEN students	Six subject teachers who teach in grades 4–12	One social pedagogue partially in the role of SENCO	One school principal, partially in the role of SENCO, working experience as a school leader at the beginning of the training 8 years

The sampling technique took into consideration that participants’ experiences and actions can provide purposeful information and build an in-depth picture about the case (Creswell, 2007).

At the beginning of the training course, both schools applied for funding to improve the learning environment at the local government. Both schools received funding and these were co-funded by the European Social Funds (ESF).

### Ethical Issues

At the beginning of the study, all participants were explained the purpose of the study and what data would be collected, used, and stored. It was also confirmed that the confidentiality of the data is guaranteed and all the data collected on paper or in digital form are kept secure. It was clarified that all data will be used only for research purposes and the results will be presented in a generalized form, following all the requirements of the ethical study which does not allow the participants to be identified. All team members gave written consent to participate in the study.

While conducting group interviews, we took into account that ethical issues may arise related to confidentiality, in particular from the point of view of the interviewees (Sim and Waterfield, 2019). Therefore, good confidentiality practice was explained to the interviewees before the group interviews. More precisely, it was clarified that different personal opinions are expected and accepted, and participants were asked not to discuss shared personal information with others. In addition, the interview questions did not address sensitive personal information.

### Data Collection Procedure

As the use of the exploratory case study method presumes to collect data in different ways and analyze them in depth (Yin, 2003), we collected data in the following stages and formats (examples of data collection is presented in Table 2):

**Table 2:** Examples of data collection.

Data collection instrument	Examples of questions/data collected
Group interviews in the middle of the training course	What are your opinions about the content and volume of the training? What are your suggestions for making the content and volume of the training course more meaningful? What are your suggestions to increase the practical value of the training? How do you evaluate your participation in in-service training as a team?
Schools’ policy documents	Description of school vision and mission School rules of procedure Description of support system for SEN students School curricula School development strategy plan
Team homework of training course	Analyzing the situation of school culture Analyzing and updating the school policy documents Mapping the learning environment and resources Mapping school staff’s training needs Analyzing school’s self-evaluation results Planning or finalizing long-term school development plan
Group interviews at the end of the training course	What school’s development activities have you carried out during the training course at your school? What development activities have you planned after the training? What have been the supporting/hindering factors in implementing the changes?
Open-ended questionnaire at the end of the training course	What has been my role as (teacher, support specialist, school leader) in implementing the planned development activities? What are the most important factors influencing team learning?
Research diary	Monitoring the participation and discussions of team members during the training course Monitoring the commitment of participants

#### Group Interviews in the Middle of the Training

After the sixth session, semi-structured school-based group interviews with both school teams separately were conducted by trainers. In choosing the group interviews, we relied on Cohen et al. (2007) explanation that group members who have worked together can support or complement each other. The purpose of the group interviews in the middle of the training course was to get feedback on the content, volume, and organization of the training course to make modifications if necessary and thereby better support schools in their development activities. Interviews were between 1 h and 1 h and 10 min in length.

#### School Policy Documents

A desktop analysis (Mason, 2002) of available school policy documents on IE was carried out in the beginning and at the end of the training course with the aim to map the preliminary situation and find out the final modifications. This method made it possible to understand how schools’ inclusive education policies have changed and are reflected in formal documents. We analyzed documents that are mandatory for schools by law and must be publicly available.

#### Team Homework Assignments of Training Course

The homework submitted during the training was purpose-built documents for the study, which provided an additional opportunity to get answers to the research questions: what development activities were carried out during the in-service training course to implement IE at the schools, and what factors affected the development activities planned and carried out in the implementation of IE. According to Gillham (2000), this method makes it possible to keep track of what the case study participants *said* and what they actually *did*.

#### Group Interviews After the Training Course

As semi-structured interviews are the most important form of interviewing in case study research (Gillham, 2000), we conducted additional group interviews at the end of the training course. The purpose of these interviews was to find out what development activities on IE schools were carried out during the training course and what were the supporting/hindering factors in implementing the changes. The duration of interviews with both schools was 45 min.

#### Open-Ended Questionnaires After the Training

Individual open-ended questionnaire as an additional method was chosen to complement the group interviews and sought further answers in particular to the second research question. Bryman (2016) and Mason (2002) suggest to use this method as it allows all participants to individually provide their personal opinion and additional information.

#### Researcher’s Diary

A research diary was kept by the first author of the study during the training course. This method helped to reflect the results of the research in a more open and honest way (Engin, 2011).

### Data Analysis

In the current case study, the form of the embedded analysis of different units was used (Yin, 1994). The preliminary situation, process, and final outcomes of schools’ developmental activities and factors affected these activities were analyzed using multiple data collection instruments.

The data analysis procedure consisted of three phases.

#### Preparation Phase

The aim of the preparation phase was to prepare data for thematic content analysis. Interviews with both school teams were recorded and transcribed in full. Schools’ policy documents, the training course homework, open-ended questionnaires, and research diary notes were documented separately by the schools. The total volume of the data was 110 pages in the first case and 108 pages in the second case.

#### Case by Case Analysis Phase

In the second phase, a thematic content analysis was conducted separately by cases as it enabled to describe the meaning of qualitative data systematically and rule guided but also in a flexible way (Schreier, 2012). All documented materials were repeatedly read with the aim to select the meaning units by the research questions. Consequently, condensed meaning units were coded, which in turn were listed in a separate file. The list of codes included the name of the code, description, and examples of the meaning units. After the coding process, the codes were grouped under subthemes and main themes. For example, the codes “changing the system of development conversation,” “monitoring individual development of students,” formed a subtheme, *supporting students*. The codes “mapping teachers’ training needs,” “in-school trainings for teachers,” formed a subtheme, *supporting teachers*. The codes “renewal curriculum,” “preparation of a development plan,” formed a subtheme, *school policy*. Finally, three subthemes formed the main theme, structural change. The two cases are described by the main themes and subthemes.

#### Multiple Case Analysis Phase

In the final phase, a cross-case analysis was conducted using qualitative meta-analysis synthesis to compare and synthesize themes and subthemes, with triangulation of findings across cases to support validity of the study (Mays and Pope, 2000). The focus was on the pattern establishment and generalizations. At the end of this phase, the analysis revealed similarities and differences of the cases and the results are presented by the main themes combined with subthemes. For example, describing what factors affected school development activities, the subthemes *development activities led by appointed leader, school leader as a member of the team, teacher as a leader among other teachers* formed the main theme “leadership.”

The initial data analysis was done by the first author. Following, all co-authors were involved in the final data analysis process and both coding and categorization decision were discussed until a consensus was reached.

In the following section, we describe the results of the data analysis on a case-by-case basis, which development activities were carried out by the schools and what factors affected it. We also present a comparison of the two cases and discuss the most important results.

## Results

### Case No. 1

#### Development Activities Carried Out During the Training Course

In the first case, a change of school leader took place 2 years prior to the training course in which the data from this study were collected. The teaching staff in this school had also changed to a large extent. Due to the increasing proportion of students with special educational needs in the school, the school leader had set a priority to improve implementing IE in their school. At the beginning of the training, discussions took place between the parties involved in the school (teachers, parents, students) about the school’s vision and values. As a result, the main principles of IE were jointly agreed and, most importantly, inclusion was considered in a broader sense, i.e., inclusion concerns all learners, not just those with special needs. A joint agreement was made at the school that special classes would not be formed for SEN students, instead IE supported by co-learning with peers in an inclusive classroom. However, if necessary, sufficient support would be organized individually or in groups. The mapping of inclusive school culture conducted during the training course revealed that not all teachers share inclusive values to the same degree and therefore the goal was to keep the development of inclusive school culture in focus among teachers and the wider school community.

To support the relationships between students in the school, a support program for students with learning difficulties and behavioral problems was implemented during the training course. Some students became support peers for other students on a voluntary basis. This was considered important, in particular, to support student-to-student friendships and to provide student-to-student assistance, but also to enhance cooperation between students and teachers. The school also joined an evidence-based anti-bullying program.

In order to support all students and to notice the individual special needs of students at an early stage, the procedure for developmental interviews with students and their parents was arranged. By the end of the training, a thorough procedure and instructions for conducting development interviews for teachers as well as parents and students were completed. The teacher who participated in the training said:

My favorite development activity was the topic of development discussions. This did not happen systematically in our school. Now we have specific guidelines and forms for collecting feedback from students and parents and documenting the developmental interview.

An analysis of the school’s SEN student support system at the beginning of the training course indicated that it is not sufficiently systematic and comprehensible to all parties. Therefore, the school team focused on updating the system for monitoring and intervening in the individual development of students, which resulted in the reorganization of the entire school support system. As a result, the principles and objectives of support were formulated, the support services provided at different levels were described, the roles of the different parties were specified, the principles of cooperation in supporting students, and the criteria for evaluating support results were defined. Under the leadership of support specialists, this was immediately implemented in the school.

In order for the renewed support system to be implemented effectively at the school level, internal training was organized for all teachers. Under the leadership of support specialists, a learning community was initiated for teachers and parents, where it was possible to discuss how to find solutions to the problems that have arisen in the involvement of students with SEN. At the same time, the need for longer-term training for teachers was mapped based on the specifics of meeting their own development needs and development goals. The school also decided to initiate a mentoring program for new teachers, and one part of this was the SEN student support system at school. In order to provide comprehensive support to teachers, support specialists also passed through the training in co-vision techniques.

As the basic document of the school’s operation is the school curriculum, the extent to which the curriculum supports the provision of quality education for children with SEN was analyzed. As a result, the school curriculum was supplemented. Firstly, the members of the school team focused on formulating minimum learning outcomes for students with learning difficulties, and secondly, the further task of the support specialists was to supplement the development of the general competencies described in the curriculum. SENCO of the school explained:

Speech therapists should look at how to achieve communication skills, the task of a social pedagogue is to develop social skills, a special educator should look at the topic of learning skills and a psychologist the topic of emotional skills. And then the school curriculum will frame these important points on how to support students in these areas.

At the end of the training, the school had prepared a new development plan for the next 3 years, which defined the following development areas: systematic and value-based management of the school; supporting the development of inspired, collaborative, satisfied and professional staff; effective cooperation with stakeholders, and creating an inclusive school environment. The participants themselves emphasized:

Since we consider inclusion in our school in a broad sense, all the planned development activities in our development plan are in fact the development activities of an inclusive school.

As can be seen from the above description, during the training course, the school was able to improve the functioning of the inclusive education system as well as to draw up a long-term strategic plan with clear objectives and specific activities.

#### Factors Affected Development Activities

The team that participated in the training had set a specific goal to reach a development plan by the end of the training course, which also defines further development directions. The school leader appointed a support specialist to lead the development activities of IE, who also performed the tasks of the SENCO at the school. In the case of the teachers selected for the team, the principle considered that they would be motivated to improve themselves in the field of IE and thus, contribute to development activities. The school leader did not take a leading role and was involved as a member of the team, and this was explained as follows:

The fact that I chose a specialist to lead the process was, in my view, the only right decision. With her knowledge and dedication, she was the real leader we were able to rely on.

All team members were committed to addressing all the topics covered during the training. It was emphasized that the involvement of different specialists working in the school in the training course increased both the cooperation between them and the cooperation at the school level as a whole. The possibility of cooperating with other schools was also considered an encouraging factor. However, participants pointed out that the implementation of IE in schools is greatly influenced by how it is supported at the national level. They mentioned a lack of state support in ensuring the availability of necessary support for learners with SEN, such as directing resources to access out-of-school counseling services, developing teacher training, improving learning environments, and creating study materials for different levels of learning.

The implementation of the planned activities was supported by the ESF co-financial support for the improvement of the learning environment and received at the beginning of the training. At the end of the training, the school had an extension of a school building, which solved the lack of space, especially in providing flexible learning opportunities and the necessary support for students with SEN. During the training, after mapping the need for support professionals, the school head found an opportunity to hire more staff of support specialists. The school’s team members were pleased with this situation, but emphasized:

However, in the implementation of the planned development activities, we will continue to see the need to contribute to the improvement of the learning environment as well as to the increase of the existing human resources. But now we face the challenge of how to use them most effectively in a context of limited resources.

The lack of time was emphasized as a critical factor in planning and implementing all activities during the training course, but the team coped well with time planning. Participants acknowledged that in a time-constrained environment, skillful time planning and consistency in adherence to the plan are important. As such, it was possible to meet with the team on a weekly basis, if necessary, conduct brainstorming with the entire staff, and contribute to the homework provided during the training course.

According to the participants’ point of view, they were also supported in planning the development activities of IE by the fact that during the training course it was possible to comprehensively address various aspects of IE and thus create a systematic approach to achieving both short-term and long-term goals. The role of trainers as external experts was considered important. The trainers’ broad knowledge of the meaningful implementation of IE, taking into account evidence-based practice and linking theory to practice, was highlighted as positive. However, participants acknowledged that there was a lack of individual school visits and counseling during the training period. Regarding the recommendations of the specialists of the regional out-of-school counseling team, it was pointed out that their decisions are often inconsistent with the school’s SEN student support system and do not support inclusive classroom practice.

In conclusion, the clearly set short- and long-term goals and SENCO’s committed leadership in promoting the key topics supporting implementation of IE covered in the training course encouraged all members collaboratively contribute to the planned activities. However, there was a need for greater state involvement in the implementation of inclusive education policies and more effective out-of-school counseling services.

### Case No. 2

#### Development Activities Carried Out During the Training Course

In the second case, the school leader had been in office for 8 years and the school team was guided by the vision and core values previously developed in the training activities. The core values reflected in the school’s documentation were openness, cooperation, and creativity. Good education is ensured for each student according to their level of development and ability-based grouping of students. At the beginning of the training, participants explained that the core values of their school reflect the nature of IE. However, during the training, the concept and meaning of IE was discussed and it was decided to set out more clearly the principle of inclusive education, according to which students with SEN generally study in the mainstream classroom and receive the necessary support. However, participants emphasized:

Providing inclusive education principle in documents and the introduction of this idea alone will not help. However, inclusion is encouraged by the continuous promotion of the organization’s culture and spirituality. We need to communicate our values both inside and outside the school.

As the school has a large number of students of different nationalities as well as students with different SEN, an evidence-based behavioral skills development program was introduced at the school to ensure the safety of all students. During the training period, the implementation of this program was extended. In addition, the school’s rules of procedure were amended to make more precise the guidelines for the behavior of all learners, including those with SEN, in different situations. The participants of the training indicated:

Such clear instructions were actually useful for other students as well. Everyone immediately had fewer problems.

In order to ensure the well-being of the teachers, one person was selected from among the teachers to mediate the problems and concerns to the management board.

To improve the necessary support for students, the practical arrangements for early detection of SEN and the availability of support in their school were analyzed and organized in such a way that there is a comprehensive system that supports all those in need. The responsibilities and tasks of the different parties, the principles of providing support, the support services provided, and the criteria for evaluating the effectiveness of IE were specified. By the end of the training, the team had changed the SEN students support procedure document in cooperation with the support specialists and teachers working at the school and made it available on the school’s website.

In order to enhance and support cooperation between teachers, a subject section on IE was launched, where teachers could exchange their experiences and provide the necessary counseling from support professionals or teachers who had participated in the training. In order to plan teachers’ individual subject training needs, as well as the inclusion needs of children with SEN, the school head drew up a matrix of teacher development needs based on school values on the one hand and the professional standard of teachers on the other. Based on this matrix, teachers can analyze their development needs in implementing IE, plan training courses, and thus shape their careers.

The team that participated in the training also analyzed the school curriculum from the point of view of inclusive study organization, and thorough changes were made to this document: the principle of IE was set and the principles of supporting children with special needs and counseling parents were brought into line with the improved system and legislation. However, the participants commented:

Now that we are streamlining our IE system, we are coming up with new ideas and therefore realizing that the curriculum needs to be constantly updated and improved. It will never be finished.

In conclusion, it can be said that during the training, the school team worked to improve the internal support of students with SEN, to organize the documents concerning the organization of IE, and to map further development needs. The aim was that the knowledge and information gathered during the training would be analyzed more thoroughly together with the entire school staff and used as a basis for compiling a new development plan.

#### Factors Affected Development Activities

The school team had set the goal of improving the organization of support for learners with SEN during the training course and defining the development goals of IE. The school leader gave the initial initiative in planning the development activities of IE to the team participating in the training course. The school leader submitted proposals both in the mapping of the situation and in the planning of activities in the phase when the need for development activities in one or another area had become clear. Once completed, the proposals were submitted by the school leader and justified this as follows:

I made a very conscious choice for my school team. It was important to me that the team included proactive support professionals and teachers from all levels of primary school. I delegated the management of this whole process to them, as they communicate most closely with both teachers and students.

The school team was motivated to deal with the set goals and the cooperation between the team members went well. At the same time, it was pointed out that not all teachers were sufficiently involved in the mapping and planning of the development activities within the school. Participants felt that not enough support was found at the local government level to improve IE. It was explained that the implementation of inclusive education has been largely an initiative of some schools themselves, but local government education officials should take the lead in creating an understanding that all schools in their area need to teach children in an inclusive way and then provide them with the necessary support. The participants of the training also pointed out that the state education policy approach to the implementation of IE also sets certain limits in terms of the planned activities.

On the one hand, the support system for learners with disabilities is too bureaucratic and non-inclusive. On the other hand, the number of new immigrants is constantly increasing. This target group is not well supported.

However, during the training period, the school received financial resources co-financed by ESF to improve the learning environment. It was decided to invest in the furnishing of the classrooms (e.g., adjustable desks, soundproof partitions, etc.). After reorganizing the support system, the number of support specialists in the school was increased. As a result, it was possible to practice the planned activities and provide more effective support to students, teachers, and parents. While at the beginning of the training SENCO’s tasks were divided between the school principal and one school support specialist, at the end of the training SENCO was replaced by a new support specialist, as the upgraded system required more time and one leader.

According to the trainees, the fulfillment of the goals set by the school both in the improvement of the existing system and in the mapping of development needs was also supported by the complex treatment of various key topics related to inclusive education during the training and exchange of experiences with other schools. However, the trainees pointed out that the time resource set its own limits and that it was not possible to contribute enough to all the planned activities. It was also acknowledged that accurate time management and adherence to it would have helped to reduce this problem. The lack of financial resources was also highlighted. The school team would have liked to recruit more teachers and assistant teachers to reduce the workload of teachers who had more students with SEN in their class.

The involvement of external expertise in the form of trainers during the planning and implementation of the school’s development activities was considered important by the school team. In addition, as expressed, co-operation with trainers could even continue after the end of the training course. Specialists from the out-of-school counseling team were expected to provide more guidance on how to organize the teaching of students with more severe special needs, as well as students without special needs, in an inclusive classroom.

In summary, a committed team was working on improving the situation related to IE. A clear leader of the work was not specified. The goals were met, and the team was motivated to improving IE at school. However, a number of obstacles were highlighted, such as a lack of resources and a lack of commitment from local and national authorities to support IE policies at school level. There was also a need to continue consulting with the external experts after the training.

### Cross-Case Analyze

#### Similarities and Differences Between the Two Cases in Development Activities Carried Out During the In-service Training Course

We compared the similarities and differences between the two cases regarding the activities that were carried out during the training and identified different cultural and structural level changes (see Table 3).

**Table 3:** Similarities and differences between the two cases in development activities on inclusive education (IE).

Main theme	Development activities carried out during the training course	Case 1	Case 2
Shaping school culture	Shaping vision and values at the beginning of the training course	+^*^	−^**^
	Involving stakeholders in shaping vision and values	+	***
	The need for further communication of values inside and outside the school	+	+
	Establishing the concept and meaning of IE	+	+
	Peer support activities among students	+	−
	Implementing evidence-based behavioral programs	+	+
	Appointment of a teacher welfare coordinator	−	+
Structural change	Changing school curricula on IE	+	+
	Composing of a new strategic development plan on IE	+	−
	Enhancing the developmental dialogue with students and parents	+	−
	Improving the support system for SEN students	+	+
	Changing the internal rules of the school	−	+
	In-school training for all teachers on IE	+	−
	Dealing with IE topics in teachers’ workshops	+	+
	Mapping the training needs of teachers and other school staff	+	+
	Preparation of a training plan for teachers and other school staff on IE	+	−
	Compiling a self-assessment matrix for teachers’ training needs	−	+

* + Activity took place;

** − No activity;

*** No data.

##### Shaping School Culture

A comparative analysis of the cases shows that schools dealt differently with the topic of vision and values during the training course. In the first case, the school had decided to start developing targeted IE just before the training course. In cooperation with all parties, the vision and values of the school were set out, including the principle of inclusive education, which became the basis for mapping the current situation and planning further development activities. The focus was to increase the capacity of the whole school to teach students with SEN in an inclusive classroom. In the second case, the vision and values were defined years earlier and their renewing was not discussed. However, the school team decided that the principle of IE and its meaning should be more clearly articulated in the school documentation, as there were no common understandings of the meaning of IE at school level. Designing a school culture for the meaningful implementation of IE remained a challenge for future development.

Evidence-based behavioral programs were used in both cases to develop good practice and ensure safety for all students. Additionally, a peer supporting program was initiated in the first case. In the second case, to ensure the well-being of the teachers, one person was selected from the teachers who was appointed as the coordinator of the well-being of the teachers and whose task was to communicate the problems and concerns of the teachers to the management.

##### Structural Change

In both cases, the training involved organizing and drafting the school’s key policy documents, although in different ways. In the first case, the priority was to develop a new development plan for the school, and this goal was met. The completed strategic document was clearly communicated, with specific targets and measurable development activities, which reflected the characteristics of an inclusive school and where the creation of high-quality learning opportunities that support the individual development of the student had been identified as the most important development activity. In the second case, the part of the school curriculum dealing with support and counseling for students with SEN was updated. Preparations of a new development plan were also started. It was emphasized that the situation of the self-assessment questionnaires, learning environment, resources and training needs conducted during the training course were mapped and analyzed by the members participating in the training course and provided a lot of valuable information, all of which needs to be discussed with the whole school staff.

In the first case, the procedure of developmental dialogue with students and parents was thoroughly addressed, as it did not work systematically at school. In the second case, it was not considered necessary to make changes to this document. According to the participants’ point of view, the procedure of developmental dialogue was well organized in their school. In both cases, the system for noticing, intervening and documenting the need for support for learners with SEN was streamlined. The school team was recognized at the local government level for this activity in the first case. In the second case, rules of procedure were amended. The amended procedures made the rules of good behavior clearer for students with SEN as well as for other students.

In both cases, in-school training was provided to support teachers. In the first case, the training was conducted by the participants for all teachers in relation to the needs mapped during the training and the revised documents. For example, it became clear that the roles of support professionals and the support system were not clear to all teachers, and in-school training was provided on the subject. In the second case, teacher-to-teacher training was provided. The teachers who took part in the training, who were selected from different school levels, shared the knowledge gained during the training in smaller study circles. In the first case, too, learning communities were initiated for teachers, but in terms of content, they aimed at solving the problems that had arisen. In addition, the school mapped teachers’ training needs, which highlighted the need to increase teachers’ knowledge and skills in three key core values related to inclusive teacher education: valuing learner diversity, supporting all learners in an inclusive classroom, and collaborative skills. As a result of the mapping of the training needs, a long-term teacher training plan was prepared to ensure the fulfillment of the goals set in the development plan. In the second case, the school head drew up a comprehensive self-assessment matrix for teachers’ training needs for IE, based on which teachers themselves mapped their training needs and then draw up an annual training plan.

In conclusion, although both schools contributed to the planning and implementation of development activities in different ways during the training, in both cases their own goals were followed, and they were achieved. In the first case, the training focused intensively on the development of all the topics covered during the training course, and a development plan was completed, setting targets for the next 3 years. In the second case, the main focus was on the mapping of development needs, on the basis of which it was planned to start preparing a new development plan after the end of the training course. However, while considering different activities, it also became visible that both schools focused more on the structural changes and somewhat less on the cultural changes.

#### Similarities and Differences Between the Two Cases About Factors Affecting School Development Activities on IE

Next, we compared the similarities and differences regarding the factors that the trainees considered important in the planning and implementation of IE development activities (see Table 4) and distinguished these across six broader main themes.

**Table 4:** Similarities and differences between the two cases factors affecting.

Main theme	Factors affecting	Case 1	Case 2
Leadership	Development activities led by appointed leader	+^*^	−^**^
	School leader as a member of the team	+	+
	Teacher as a leader among other teachers	+	+
Commitment and contribution	The commitment of the school team involved in the training	+	+
	Motivation of the school team involved in the training	+	+
	Clearly stated goals	+	+
	Commitment and contribution of all staff	−	−
	The need for contribution at national level	+	+
	The need for contribution at local government level	−	+
Collaboration	Efficient cooperation between the members of the training team	+	+
	Cooperation with all teachers at the school	−	−
	Out of school cooperation	−	−
	Cooperation with other schools	+	+
System-wide approach	Systematic mapping of the current situation in the implementation of IE	+	+
	Prioritization of development activities on IE	+	+
Resources	The need for more time	+	+
	Effective time planning	+	−
	Resources for improving the inclusive learning environment	+	+
	The need for financial resources to hire more staff	−	+
External expertise	Knowledge and skills of trainers	+	+
	Evidence and research based approach	+	+
	Linking theory into practice	+	+
	The need for individual school visits and counseling during the training course	+	−
	The need for continuing cooperation between schools and trainers after the training	−	+
	The need for adequate support of specialists in out-of-schools counseling centers	+	+

* + Factors appeared.

** − Factors not appeared.

##### Leadership

The schools had organized the leadership of development activities differently. In the first case, a specific leader, SENCO, working at the school, was appointed to lead the whole development process. The school leader was an active member of the team and participated in the process of mapping development needs as well as planning improvement activities. In the second case, there was no specific leader in the activities carried out during the training course. Teachers and support professionals who participated in the training course mainly contributed to the improvement of the support system for students with SEN. The school leader took responsibility for ensuring the professionalism of teachers, such as conducting a self-assessment questionnaire among all teachers and mapping teachers’ training needs.

In both cases, it appeared that teachers had become carriers of inclusive thinking and practice and there for also leaders for their colleagues within the school.

##### Commitment and Contribution

Both schools were motivated to participate in the training course and thus, to enhance IE arrangements so that all students would be supported in an inclusive classroom. In the first case, in addition to immediate actions to improve the efficiency of the support system, a long-term strategic development plan was drawn up during the training. The other team aimed to map out the areas that need to be thoroughly developed, which would support them in drawing up a development plan after the training course.

In both cases, all team members participated in the training activities as well as in the homework assignments. Participants emphasized that taking responsibility was voluntary and that everyone contributed to the activities in which they felt a strong commitment. This was also supported by the fact that at the beginning of the training course both schools had clear goals and objectives, which they want to achieve by the end of the training course. At the same time, in both cases it was stated that the involvement of the whole school staff in the development process still needs to be improved. It was also pointed out that not all teachers have an understanding of the meaning and importance of inclusive education.

At the same time, it was emphasized in both cases that they perceived little contribution from the state to the implementation of inclusive education policies and to the support of schools in the implementation of IE. It was pointed out that there is a lack of a clear vision and goal at the national level on how to make the whole education system more inclusive and gaps in legislation were also observed. In the second case, the lack of commitment of local authorities to promoting inclusive education at regional level was also highlighted.

##### Collaboration

The cooperation between the members of both teams who participated in the training course went well. It was pointed out that working together during the training strengthened the relationships between the team, which in turn had a positive effect on the cooperation within the school with other teachers who have readiness to teach in an inclusive classroom. At the same time, in both cases, there was a greater need to involve parents and the community in planning development activities. Cooperation with other schools was highly valued as the exchange of different experiences during the training provided lots of ideas on how to solve different problems and what aspects to pay attention to in the school’s development activities.

##### System-Wide Approach

In both cases, it was considered important that the training addressed the most important issues related to the development activities of an inclusive school in an integrated way. It was pointed out that linking theory to practice helped to create a broad-based background for mapping the real situation and planning development activities.

##### Resources

Both schools found opportunities to improve their school environment and recruit additional staff. However, school No. 2 pointed to a greater need for financial resources to implement its plans. The lack of time was emphasized by both teams. On the one hand, this was due to the fact that the school development process was very time-consuming, and on the other hand, schools were overburdened with activities due to the COVID-19 crisis. However, in the first case, the team was able to plan their time very carefully for both completing homework assignments and development planning. Although, they admitted that it was very burdensome and at times they acted within their capabilities. In the second case, regular meetings were not planned or the planned activities were postponed due to lack of time.

##### External Expertize

In both cases, it was considered important that an expert from outside the school be involved in planning and developing the development activities of IE, who would be able to point out the mapping of different key areas and their interrelationship. It was pointed out that the in-service training program developed at the university provided a good opportunity for this. The research- and evidence-based approach to supporting school self-development activities was considered very important. Linking theory and practice during the training course was also highlighted as a supporting factor. At the same time, the need for more individual meetings with trainers during the training course was acknowledged. It was also pointed out that cooperation with trainers could continue after the training course. As the closest advisory experts at the school level are specialists from the regional out-of-school counseling team, it was criticized that their advice did not always support schools how to teach students in an inclusive classroom.

In conclusion, participation in the training course as a team, the commitment and specific goals of the team, cooperation with other schools, the complexity and coherence of the topics covered in the training course were seen as supporting factors in schools’ development process. The lack of time and involvement of the parties of the whole school was an obstacle. The low contribution of the state and local government to the issue of inclusive education was seen as an out of school hindering factors. A need for individual counseling was reported by trainers both during and after the training. Regarding the practice of an inclusive classroom, the trainees felt little support from the specialists of the out-of-school counseling centers. On a more general perspective, both schools pointed out more supportive factors and some hindering factors regarding school level factors, while regarding the region and state level concerns and hindering factors were voiced more than the supportive factors.

## Discussion

The aim of the current study was to explore how long-term in-service training for school teams (teachers, support specialists, school leaders) influenced schools’ development activities in the implementation of IE and what factors affected it in school teams’ perspective.

During the training course, schools carried out several short-term development activities according to their school’s needs. The focus was on activities related to shaping school culture, as well as updating policy documents on IE in the school and thus enhancing support for students and teachers. Longer-term goals for further activities were also set. In one case, a school development plan was drawn up for the next 3 years, and in another case it was decided to start working after the training. Thus, the training had a positive effect on the development activities of schools in the field of IE. As school self-development is considered to be one of the most important criteria for removing barriers to the implementation of inclusive education (Hadfield and Ainscow, 2018; Ainscow, 2020), in-service training for school teams is a good opportunity to support them in this process. Moreover, restructuring the school policy and practice can improve the learning outcomes of all students (Persson, 2013).

The results of the case study showed that the development activities planned and carried out during the training course depended to a large degree on how the school had understood the concept of IE. If the school understood IE to mean teaching all students together in an inclusive classroom, the mapping of development needs, and planned development activities, also focused on how to increase the capacity of the whole school to put IE into practice. Although the principle of IE has been one of the basic values of education policy in the Estonian context for more than 10 years, the meaning of the concept of IE is still understood very differently (Kivirand et al., 2020) and this was also confirmed by this study. The same trend is highlighted in several studies in other countries (Hardy and Woodcock, 2015; Cameron, 2017), and our study indicates again the importance of reaching the agreement regarding the concept of IE in the Estonian context. Moreover, in the Estonian context, the most important features of IE are considered to be social inclusion and high-quality education for all learners (Ministry of Education and Reseach, 2021), i.e., the focus is on creating opportunities for students with SEN to study in mainstream schools. However, it is common practice that students with more severe SEN spend most of their time, either partially or continuously throughout the school year, in a special class or in a smaller group. Schools that use the practices described above identify themselves as inclusive schools. Black-Hawkins and Florian (2021) have also pointed out that schools that contribute to providing support and learning opportunities for SEN students in mainstream schools consider themselves inclusive schools. Therefore, addressing the different characteristics of an inclusive school culture during the in-service training course is important to change what we mean by the concept of IE. However, shaping shared values across the school is a long and complex process. In order for the values and the principles of IE developed by the team to be more widely recognized among the entire school staff, more activities could be planned for further training that would involve the whole school staff in shaping the vision and values. More emphasis should also be placed in initial teacher education on how to put IE into practice in a meaningful way. This, in turn, would provide a good starting point to engaging in a constructive dialogue in society as a whole in order to remove barriers between two somewhat opposing discourses, “inclusion for some” or “inclusion for all,” as Leijen et al. (2021) have highlighted in their study.

The changes planned and carried out during the training in the policy and structure of the school (see Table 3) were greatly influenced by the existence of a specific leader. As the school leader in the first case had chosen a specific leader to lead the whole process, they were able to plan time more effectively throughout the process and meet the short-term and long-term goals set. Appointing a support specialist, who is also in the role of SENCO at the school, to lead the change in IE is one way to map systematically all development needs and plan future activities on IE. As SENCO is the most involved with teachers and the school’s support team on a day-to-day basis, she perceives the need to support teachers in teaching SEN students in an inclusive classroom. In this way, it is possible to implement certain innovations immediately and thus also change the role of support professionals in supporting both students and teachers. Also in the international context, the focus is on the changing role of support professionals, especially in supporting teachers and introducing collaborative teaching practices (Perez et al., 2017; Florian, 2019). The results of this study revealed that a dedicated and professional leader encouraged and motivated all team members to contribute to all planned development activities. Even more, teachers who participated in the training course became carriers of IE values and communicated about practical solutions for other teachers in the school. According to Mangope and Mukhopadhyay (2015), cooperation between teachers in turn promotes their professional self-development. The participation and contribution of school leaders throughout the training was also an important positive factor. School leaders saw their role in implementing inclusive education primarily in communicating inclusive philosophy and values both within and outside school, facilitating collaboration between different actors, providing resources to overcome barriers, and creating opportunities for teachers’ professional development. The role of the school leader in shaping an inclusive organization has also been emphasized in several studies (Harris and Jones, 2017; Murphy, 2018; Lambrecht et al., 2020). Khaleel et al. (2021) have also found that the role of the school leader is largely divided into two groups: creating an appropriate environment for internal school-based activities, and creating a social, academic, and emotional atmosphere; and out-of-school activities such as communication with parents and regional policy makers. Thus, the participation of school principals in in-service training, which deals with the planning of cultural and structural changes throughout the organization, is very important for their realization.

The study showed that the participation of different school teams in the in-service training course was also a positive factor. Sharing experiences with other schools made it possible to learn from each other and thus enhanced the school’s development activities in the context of their own school and enabled to create a basis for further collaborative activities. The effectiveness of inter-school collaboration in the process of development activities in IE has also been proved in several other studies (see Ainscow et al., 2006; Ainscow, 2015). Thus, more collaborative inter-school learning communities should be created at the local level to enhance the development of meaningfully inclusive schools through the process of self-development activities. Moreover, as members of the school team who participated in the training course saw out-of-school factors as the main hindering factors affecting the implementation of IE, such as the lack of commitment and contribution from the state and local government, more opportunities should be found for wider cooperation. The creation of regional learning communities, involving representatives of the state, local government, schools, and universities, would help to develop shared understandings of the meaning of the concept of IE and to create different models for putting it into practice in the context of their own country. Good examples of this can be found in long-term studies in the UK (see Ainscow et al., 2020). This would also make it possible to better understand the role of the state and local government in a meaningful implementation of IE. This does not mean only more financial support, but it is essential to establish proven practices based on research in the context of a particular country. It would also help to approach the development of IE both at the organizational level and in the education system, as Kinsella, 2020 has emphasized in his study. Therefore, even when planning further training, it could be considered whether and how representatives of the state and local governments could be involved at some stage in the training course, in order to initiate a dialogue between the various parties and thus support the schools more effectively.

In conclusion, the participation of different school-level key professionals in long-term in-service training creates a good and broad-based opportunity for school self-development activities in the field of IE. At the same time, it is important that schools are supported at national and local level in this journey.

## Conclusion

The results of this study showed that long-term in-service training course for school teams (teachers, support professionals, school leaders) supported schools in planning and implementing IE development activities. In terms of development activities, two main themes were distinguished: activities related to shaping school culture and structural change. Regarding inclusive school culture, the main focus was on developing a vision and value for a meaningful implementation of the concept of IE. However, more activities took place in the context of structural change, such as reforming school policies, renewing the student support system and support for the professional development of teachers. Among the supporting factors within the school, the clearly set goals to be achieved, the commitment of the team that participated in the training course, the contribution of all participants, and good co-operation between them came up. The lack of time for cooperation within the school and the involvement of all school staff in the planning of development activities turned out to be the most essential hindering factors within the school. The out-of-school supporting factors were considered by the trainees to be a system-wide approach to all topics, the integration of theory and practice during the training course, and cooperation with other schools. The main out-of-school hindering factors were the commitment and contribution of the state and local government to the development of an IE system and the lack of evidence-based research in the field of IE in the context of their own country.

Although the study provided a good overview of the effects of team training in the planning and implementation of school self-development activities in the field of IE, we would also like to point out some limitations. Firstly, due to the COVID-19 emergency, we had to hold half of the sessions *via* Zoom and it was not possible to organize individual school counseling sessions in schools, which would have made it possible to increase the effectiveness of training in the school as a whole. Secondly, school observation as additional data collection method would have provided better information to triangulate the analysis of results, but this was also not possible in the COVID-19 situation. Further research is needed to examine longitudinally how the innovative activities implemented in the in-service training course for school teams influence the social and emotional well-being and academic achievement of all students (SEN and non-SEN students) and satisfaction of parents.

## Data Availability Statement

The raw data supporting the conclusions of this article will be made available by the authors, without undue reservation.

## Author Contributions

TK contributed to all elements of the research, design and conducted an in-service training course, collected and analyzed the data, described the results, and compiled a discussion. ÄL and LL contributed to the research design, final data analysis process, and editing and reviewing the manuscript. All authors contributed to the article and approved the submitted version.

## Funding

This research was funded by EEA Financial Mechanism 2014–2021, Higher Education in Baltic Research Program, Project Contract No 36.1-3.4/289.

## Conflict of Interest

The authors declare that the research was conducted in the absence of any commercial or financial relationships that could be construed as a potential conflict of interest.

## Publisher’s Note

All claims expressed in this article are solely those of the authors and do not necessarily represent those of their affiliated organizations, or those of the publisher, the editors and the reviewers. Any product that may be evaluated in this article, or claim that may be made by its manufacturer, is not guaranteed or endorsed by the publisher.
